# *Gazania rigens* (L.) gaertn leaf extract-inspired innovative synthesis of silver nanoparticles and promising applications as antibacterial and cytotoxic agents

**DOI:** 10.3389/fchem.2026.1774851

**Published:** 2026-04-07

**Authors:** Afrah E. Mohammed, Reham M. Aldahasi, Hessa O. Aldraiwiesh, Ishrat Rahman, Munirah M. Alhammadi, Najah Albadari, Mohammed Khaled Bin Break, Sahar S. Alghamdi, Kawther Aabed

**Affiliations:** 1 Department of Biology, College of Science, Princess Nourah bint Abdulrahman University, Riyadh, Saudi Arabia; 2 Microbiology and Immunology Unit, Natural and Health Sciences Research Center, Princess Nourah bint Abdulrahman University, Riyadh, Saudi Arabia; 3 Department of Basic Dental Sciences, College of Dentistry, Princess Nourah bint Abdulrahman University, Riyadh, Saudi Arabia; 4 Department of Pharmaceutical Chemistry, College of Pharmacy, University of Ha’il, Ha’il, Saudi Arabia; 5 Medical and Diagnostic Research Centre, University of Ha’il, Ha’il, Saudi Arabia; 6 College of Pharmacy, King Saud bin Abdulaziz University for Health Sciences (KSAU-HS), Riyadh, Saudi Arabia; 7 Medical Research Core Facility and Platforms, King Abdullah International Medical Research Center (KAIMRC), Ministry of National Guard Health Affairs, Riyadh, Saudi Arabia; 8 Ministry of National Guard-Health Affairs, Riyadh, Saudi Arabia

**Keywords:** biological activity, characterization, cytotoxicity, Gazania rigens var. uniflora, green synthesis, phytochemicals, silver nanoparticles

## Abstract

**Introduction:**

Silver nanoparticles are potent nanomaterials with significant applications in combating microbial infections and cancer. In this study, the extract of Gazania rigens var. uniflora was evaluated as a biogenic agent for the synthesis (G-AgNPs), and their biological activity was systematically assessed. Additionally, association of the antibiotic ampicillin and biogenic silver nanoparticles was developed to explore potential additive antibacterial effects, which has not been previously reported for this species.

**Methods:**

Silver nanoparticles were synthesized using a combination of the plant leaf extract and silver nitrate solution. Several characterization techniques, including dynamic light scattering (DLS), transmission electron Microscope (TEM), energy-dispersive X-ray spectroscopy (EDX), and Fourier transform infrared (FTIR) spectroscopy, were employed to analyze the synthesized nanoparticles.

**Results:**

DLS results indicated the presence of spherical, monodispersed nanoparticles with a mean diameter of 135.4 nm and a polydispersity index of 0.223, while TEM revealed sizes ranging from 14 to 44 nm. FTIR and EDX analyses confirmed the presence of phytochemical capping agents and elemental silver, supporting successful green synthesis. Both biogenic silver nanoparticles and ampicillin-associated G-AgNPs demonstrated antibacterial activity against Staphylococcus aureus, Streptococcus mutans, and Escherichia coli, with the ampicillin-associated G-AgNPs exhibiting a partial additive effect. Cytotoxicity assays demonstrated that G-AgNPs exerted significantly higher toxicity toward the breast cancer cell line MDA-MB-231 compared to non-cancerous breast epithelial cells.

**Discussion:**

Overall, this work distinguishes itself by integrating detailed physicochemical characterization with both antibacterial and anticancer evaluations, as well as by examining antibiotic-associated G-AgNPs synthesized using G. rigens. These findings support the potential biomedical relevance of G. rigens-derived AgNPs and justify further mechanistic and in vivo investigations.

## Graphical Abstract

**Figure:**
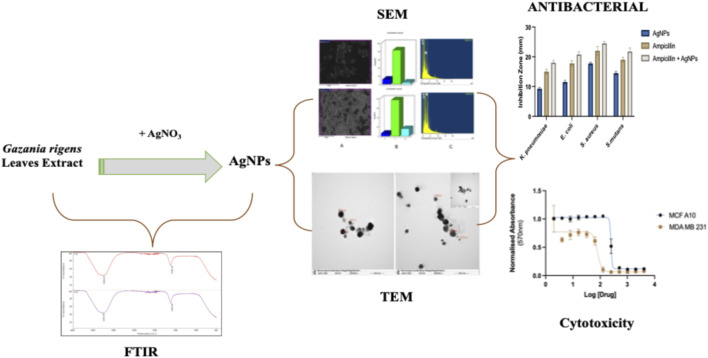

## 1 Introduction

Silver nanoparticles (AgNPs) are highly regarded for their extensive biomedical applications, including their antimicrobial and cytotoxic properties. Consequently, researchers are interested in developing AgNPs with desirable properties through eco-friendly approaches. The potential of AgNPs is mainly affected by their size and morphology, which are influenced by the methods of their synthesis. The synthesis of AgNPs using botanical extracts, microorganisms, and biomolecules is a subject of extensive research. The characteristics of nanoparticles can vary based on the biological agent used in the synthesis process. Therefore, it is crucial to develop novel methods for producing AgNPs with unique properties to advance the development of nanomaterials with a spectrum of biological functions. The properties of AgNPs relative to their bulk origin are expected to increase their application potential (Bruna et al., 2021; Yaqoob et al., 2020). NPs have emerged as versatile tools in biomedical applications, including targeted drug delivery, antimicrobial therapies, and wound healing, due to their small size, high surface area, and unique physicochemical properties (Lakkim et al., 2023; Lekkala et al., 2024; Lekkala et al., 2025).

The emergence of microorganisms exhibiting resistance to multiple drugs and cancer cells unresponsive to chemotherapy presents significant healthcare challenges. There is an urgent need to identify and develop safe and efficient agents to mitigate these problems. As an antibiotic and chemotherapy substitute, AgNPs could be one of the new antibacterial agents for treating infections (Lee et al., 2019). Recently, Krishnan et al. (2020) conducted an extensive review of silver-based nanomaterials and explored their utility in enhancing wound healing processes. Kovács et al. (2022) also reviewed the ability of AgNPs to initiate cancer cell death via various mechanisms, including their high accumulation in cancer tissues, interactions with cellular components, and the activation and modulation of several signaling pathways, as well as their induction of oxidative damage.

The effectiveness of AgNPs is often attributed to their well-recognized antioxidant properties. These properties enhance their function as efficient scavengers of reactive oxygen species (ROS), by-products of oxidative stress arising from various metabolic processes associated with human diseases (Rai et al., 2021). It could also be related to biogenic AgNPs being capped with biomolecules that enhance their compatibility with biological systems and add additional functionality (Mujaddidi et al., 2021). The conjugation of biogenic AgNPs and antibiotics has recently been investigated in a trial to enhance AgNP activity and reduce the development of antimicrobial resistance to known antibiotics. (Halawani et al. (2020) reported on the efficiency of cefotaxime-conjugated AgNPs against cefotaxime-resistant MRSA and *E. coli*. Our previous publications also investigated AgNPs as antibacterial and cytotoxic agents; a key finding was that distinct NP characteristics and activity levels are achieved using different plant types (Aabed and Mohammed, 2021b; Mohammed and Al-Megrin, 2021; Mohammed et al., 2021). In addition, the characteristics of AgNPs, such as their morphology and size, can be influenced by the specific biomolecules in each plant extract. These biomolecules play a crucial role in the reduction process from (Ag^+^) to (Ag^o^) and also affect the duration of AgNP nucleation (Krishnan et al., 2020). In addition, plant biomolecules, such as flavonoids, quinones, and alkaloids, used to synthesize the nanoparticles (NPs) also bind to the surface of the metal ions and could be a key determinant of the biological selectivity of the biogenic NPs (Mohammed et al., 2023).

To expand the search for NPs with potent antibacterial and cytotoxic properties, we used *Gazania rigens (L.) Gaertn. leaf extract*as a biological agent to synthesize AgNPs. *G. rigens* is a flowering plant (Asteraceae family) grown for landscapes and gardens (Nia et al., 2015). It is a known medicinal plant with antioxidant and hepatoprotective potential (Elkhayat, 2016), which could be related to its richness in phytochemicals such as phenolic acids and various flavonoids (Desoukey et al., 2016). Therefore, *G. rigens* was selected in this study as an eco-friendly, phytochemically rich plant source (Shahzadi et al., 2021) for the green synthesis of AgNPs, to explore its unexplored antibacterial and cytotoxic potential. The active constituents in the extract play a crucial role in reducing metal ions and facilitating their transformation into NPs. A recent study highlighted the use of *G*. *rigens* in Cu-Ni bimetallic NP fabrication and its activity as an antioxidant and catalytic agent that reduced the methylene blue dye (Younas et al., 2021). The extract from *G. rigens* was effective as a biogenic agent for synthesizing AgNPs, exhibiting potent antioxidant capability and high efficiency for removing hazardous organic dyes such as congo red and methylene blue (Shahzadi et al., 2021). In these reports, the biological evaluation of *G. rigens*-derived nanoparticles was largely limited to non-biomedical applications.

In contrast, numerous studies have demonstrated the antibacterial and anticancer potential of metal nanoparticles synthesized using different plant species-particularly against Gram-positive and Gram-negative bacteria, fungi and various cancer types, including breast cancer (Abd-Elhamed et al., 2025; Ahirwar et al., 2024; Kumari et al., 2021; Mohammed et al., 2022). Further, macrophage responses to AgNPs are known to involve metabolic reprogramming pathways (Zhang et al., 2024); however, whether *Gazania rigens*-derived silver nanoparticles affect immune-related responses through these mechanisms remains unknown. Therefore current study evaluates the antibacterial and cytotoxic potential of green-synthesized *G. rigens* silver nanoparticles as an initial step toward addressing this gap. The potential of *G. rigens-*based AgNPs to enhance antibacterial activity through association with conventional antibiotics, such as ampicillin, has not been previously examined. Addressing these gaps is critical for understanding whether green-synthesized AgNPs from *G. rigens* can offer dual antibacterial efficacy and cancer selectivity under *in vitro* conditions.

Therefore, our current investigation is pivotal in determining *G. rigens* AgNP’s (G-AgNPs) potential for further research and development in cancer and bacterial infection drug development studies. Firstly, we aimed to use *G. rigens* var. *uniflora* extracts as a biogenic agent in fabricating AgNPs and performing characterization using Dynamic light scattering (DLS), transmission electron microscope (TEM), Energy-dispersive X-ray spectroscopy (EDX), and Fourier transform infrared (FTIR) approaches. Secondly, to determine biological activity, the agar well diffusion method and MTT tests were used to screen for antibacterial and cytotoxic efficacy and potency, and to assess the antibacterial effect of ampicillin-associated biogenic G-AgNPs (Amp-G-AgNPs).

## 2 Experimental Details

### 2.1 Plant materials

*Gazania rigens* var. *uniflora* leaves were collected in September 2020 from a 4-week-old plant from the Royal Commission for Riyadh City Nursery in Riyadh, Saudi Arabia. Dr. Mudawi M. Nour identified and verified the plant. The collected leaves were cleaned, and the dried leaves were milled in a machine (IKA Werke GmbH & Co., Staufen im Breisgau, Germany) until a fine powder was obtained. The powdered leaves were stored in plastic bags at normal room conditions until further use.

### 2.2 Bio-fabrication of AgNPs using Gazania rigens var. uniflora leaves

Distilled water (100 mL) was added to *G. rigens* leaves powder (2 g) to prepare the aqueous extract (2 g/100 mL) by heating at 90 °C for 20 min under constant stirring. Such conditions were selected following preliminary trials aimed at achieving rapid nanoparticle formation with stable optical properties and minimal aggregation. After cooling to room temperature, the extract was filtered through 125 mm diameter Whatman No. 1 filter paper to remove insoluble plant residues. The aqueous filtrate was standardized by maintaining a constant plant-to-solvent ratio, extraction temperature, and extraction time across all batches. The extraction yield was estimated by evaporating a known volume at room tempreture. The standardized filtrate was stored at 4 °C and used within 48 h for silver nanoparticle synthesis to ensure consistency in phytochemical composition and reducing capacity.

High-purity crystalline silver nitrate (AgNO_3_) powder (99.5%) was used. From the plant extract solution, 5 mL was added to 95 mL of 1 mM silver nitrate solution and re-heated under the same conditions. The plant-sliver nitrate solution was stored at room temperature and covered with aluminum foil to keep it away from light until a stable color had developed. The reaction was carried out under continuous magnetic stirring to ensure homogeneity and proceeded at the native pH of the plant extract, without pH adjustment or addition of external salts, in order to maintain consistent ionic strength. The reaction mixture was maintained under dark conditions by covering with aluminum foil and stored at room temperature until a stable dark coloration developed, indicating nanoparticle formation.

Completion of nanoparticle formation was confirmed by stabilization of the surface plasmon resonance peak in UV–Vis spectra and the colour change to dark. The synthesis was independently repeated three times, yielding reproducible spectral and particle size profiles. Nanoparticles were recovered by centrifugation at 12,500 rpm, a speed sufficient for effective sedimentation of nanoscale silver particles. Pellets were washed repeatedly with distilled water to remove unbound phytochemicals prior to characterization.

### 2.3 Properties of the bio-fabricated AgNPs

Several techniques characterized the G-AgNPs. A spectrophotometer (© Shimadzu Corporation, UV-1800) was used to assess the Ultraviolet-visible (UV-Vis) absorption profile between 300 and 500 nm which encompasses the characteristic surface plasmon resonance (SPR) band of silver nanoparticles. Distilled water was used as a blank for baseline correction prior to measurements. A DLS system with a zeta sizer (NANO ZSP, Serial Number: MAL1034318, ver 7.11, Malvern Instruments Ltd., Malvern, United Kingdom) was employed to analyze the hydrodynamic size and potential. Measurements were performed in aqueous dispersion (deionized water) at room temperature, without added buffering agents, to assess the intrinsic colloidal stability of the nanoparticles.

TEM (JEM-1011, JEOL, Tokyo, Japan) detected the NPs distribution and morphology at 80 kV voltage. Scanning electron microscopy (SEM) (JEOL JED-2200 series) reported the EDS where NPs’ surface morphology and elemental composition was done. SEM images were acquired using instrument-calibrated magnification, with scale bars generated by the microscope’s internal calibration system. Fourier transform infrared (FTIR) spectroscopy was conducted using a Spectrum 100 spectrometer (PerkinElmer, United States) in the range of 450–3,500 cm^-1^ to identify functional groups associated with phytochemicals in G. rigens leaf extract and their involvement in naoparticle stabilization.

### 2.4 Antibacterial assessment of G-AgNPs

The antibacterial sensitivity test was conducted by performing the agar well diffusion assay as an initial qualitative screening assay method to assess antibacterial susceptibility, rather than as a quantitative comparison with conventional antibiotics on gram-positive (*Streptococcus mutans* and *Staphylococcus aureus*) and gram-negative (*Escherichia coli* and *Klebsiella pneumoniae*) bacterial strains, which were obtained from the Bio-house medical lab (Riyadh, Saudi Arabia). Their sensitivity to the *G. rigens* leaves extract, and the bio-fabricated G-AgNPs were tested. Microbial suspensions were prepared in saline at 0.5 McFarland (1.5 × 10^8^ CFU/mL). Strains were cultured individually on agar plates, and wells were made. After the plates dried, the tested agents were added. In a sterile environment, 40 μL of each G-AgNP (1 mg/mL) and *G. rigens* leaf extract (2g/100 mL) were added separately to each well. The agar plates were later incubated at 37 °C for 24 h, and the area around each well (zones of inhibition) was subsequently measured. Distilled water served as negative controls, while ampicillin was included as a positive control.

Furthermore, the G-AgNPs were tested against the bacterial strains to first determine the minimum inhibitory concentration (MIC) and then the minimum bactericidal concentration (MBC) using a 96-well plate nutrient broth (NB) microdilution method MIC in independent triplicates. The G-AgNPs were prepared at 0.031, 0.062, 0.125, and 0.250 mg/mL, and the bacterial strains were prepared at 2.5 × 10^5^ CFU/mL. For MIC and MBC detection, 10 μL of each strain was added to 0.35 mLNB and treated individually with G-AgNPs from each prepared concentration. Tubes were incubated for 24 h at 37 °C. Bacterial inoculum in NB was used as a negative control. MICs had the lowest bacterial growth at the lowest G-AgNP concentration (low turbidity), and the MBC values corresponded to the G-AgNP concentrations with no turbidity in the test tubes. Furthermore, broths with no turbidities were sub-cultured in fresh nutrient agars without antimicrobial agents to confirm the absence of microbial growth. MBC/MIC ratios were calculated to determine the effect of the G-AgNPs against the tested bacteria according to Clinical and Laboratory Standards Institute (CLSI) guidelines (CLSI, 2016). All experiments involving *Klebsiella pneumoniae* and *Streptococcus mutans* were conducted in a biosafety level-2 laboratory.

### 2.5 Formulation and application of ampicillin-G-AgNP conjugates (amp-G-AgNP)

A one-pot reaction was used for fabricating Ampicillin-nanocomposites, according to Fan et al. (2019), with a slight modification. One mL of ampicillin solution (1 mg/mL) was added to 1 mL of G-AgNPs solution (1 mg/mL), gently mixed on a vortex, and subsequently stored for 48 h under shaking in dark conditions at room temperature. The mixture was centrifuged, and the pellet containing the Amp-G-AgNP was washed. Ampicillin was incorporated with AgNP using a one-pot co-assembly approach. The resulting formulation represents a surface-associated ampicillin-AgNP. Finally, a 1 mg/mL solution of Amp-G-AgNPs was prepared. The antibacterial activity of the Amp-G-AgNPs was assessed using the well diffusion method described in section 2.4. All experimental tests were performed in three replicates.

### 2.6 Anti-tumor assessment of G-AgNPs

Cytotoxic potency of the G-AgNPs was determined in a triple negative breast cancer cell line (MDA-MB-231) and a non-cancerous breast epithelial cell line (MCF-10A) using the MTT assay. Cells were used at low passage numbers (<20) and seeded at density of 5 × 10^4^ cells/well in a 96-well plate reaching approximately 70%–80% confluence at the time of treatment. Cultures were maintained at 37 °C and 95%/5% (humidified air/CO_2_). After 24 h, the media was replaced with DMEM (Phenol Red-Free) supplemented with 0.5% fetal bovine serum (FBS), and the cells were incubated with the G-AgNPs for 48 h where Nanoparticle suspensions were freshly prepared and briefly sonicated prior to exposure to ensure homogeneous dispersion. To minimize interference associated with nanoparticle absorbance in the MTT assay, nanoparticle only blanks were included and subtracted from sample absorbance values.

Finally, the media was aspirated, the cells washed, and the MTT cytotoxic assay was completed, measuring the absorbance at 570 nm using a Spectra Max microplate absorbance reader (Molecular Devices, San Jose, CA, United States) (Gill et al., 2016). Mitoxantrone was used as a positive control to confirm assay performance responsiveness.

### 2.7 Statistical analysis

Data represent three experimental replicates and are reported as mean ± SD. The one-way analysis of variance (ANOVA) was performed using Prism 9.1 software (GraphPad Software Inc., La Jolla, CA, United States) for comparative analysis to detect variations among tested agents. Prism 9.1 was also used for the generation of dose-response curves and determination of IC_50_ values. A significant difference between data sets is determined by P values, *P < 0.0001*, *P < 0.001*, or *P < 0.01*.

## 3 Results and discussion

### 3.1 Biosynthesis and characterization of G-AgNPs

**FIGURE 1:**
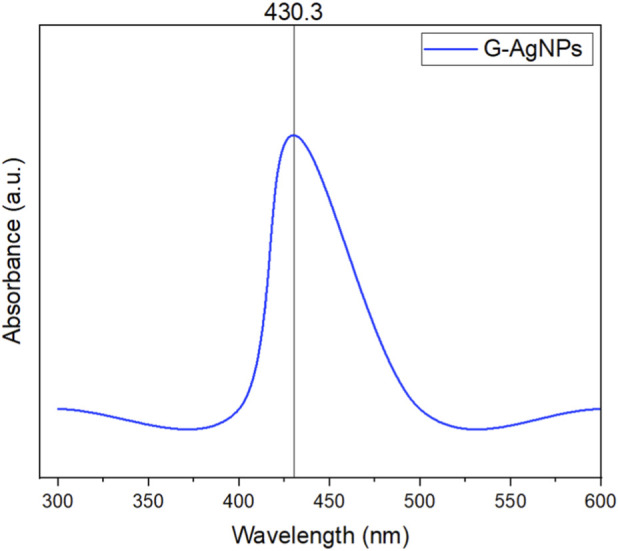
UV-Vis spectrum of G-AgNPs.

**FIGURE 2:**
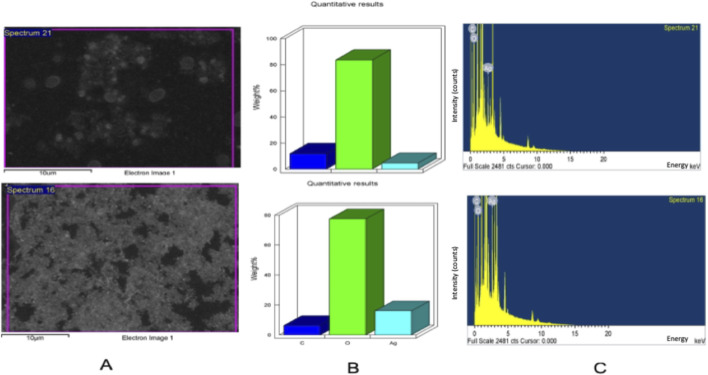
Elemental and morphological characterization of biogenic silver nanoparticles (G-AgNPs). **(A)** SEM micrographs showing the surface morphology and distribution of G-AgNPs. **(B)** EDX quantitative elemental analysis illustrating the relative weight percentages of carbon **(C)**, oxygen (O), and silver (Ag), confirming the predominance of silver with minor contributions from organic capping constituents.

The current research employed an environmentally friendly approach for AgNPs fabrication using young leaves of *G. rigens*, as they accumulate metabolites more than older ones (Qaderi et al., 2023), which are vital for the reduction process. Mixing plant extract with Ag ions led to a complete color change from light yellow to dark brown after 2 hours, indicating plasmon vibration excitation on the surface of the fabricated NPs. Various studies utilizing plant extracts have reported the same color transformation as the first sign for bioconversion of Ag^+^ to Ag^0^ (Aabed and Mohammed, 2021a; Anandalakshmi et al., 2016; Mohammed and Al-Megrin, 2021; Mohammed et al., 2021; Mohammed et al., 2021). The fabricated biogenic G-AgNPs were characterized using various techniques to confirm their formation and report their morphology as summarized in Table 1. The optical properties of G-AgNPs can provide initial information about the NPs, including their size distribution, surface morphology, and characteristics, as well as identify the reducing agents (Abou El-Nour et al., 2010). Testing of the bio-fabricated G-AgNPs in a UV spectrophotometer revealed an absorbance reading of 430.3 nm, as shown in Figure 1. Observed peaks indicate the properties fitting to NPs transition of electrons (Mie et al., 2013) which consistent with the surface plasmon resonance of AgNPs, indicating nanoparticle formation. EDX analysis for the biogenic G-AgNPs (Figure 2) confirmed spherical NPs and the existence of the elemental Ag at the 3 keV absorption peak that could be related to surface plasmon resonance. However, two additional signals for oxygen and carbon were observed, likely from the phyto-molecules in the *G. rigens* leaf extract that comprise the outer layer of the G-AgNPs, possibly highlighting their role in the reduction process (Khan et al., 2022). Identical results were also observed when Ag and Au were fabricated using *Abelmoschus esculentus* flower extract and the *Capsicum chinense* leaf extract (Devanesan and AlSalhi, 2021; Lomelí-Rosales et al., 2022).

**TABLE 1:** Summary of physicochemical characterization of G-AgNPs synthesized using *Gazania rigens* leaf extract.

Characterization technique	Parameter	Result
UV–Vis spectroscopy	SPR peak (λmax)	430.3 nm
TEM	Particle size	14–44 nm
	Morphology	Spherical/quasi-spherical
DLS size	Hydrodynamic diameter	135.4 nm
	Polydispersity index (PDI)	0.223
Zeta potential	Surface charge	−13 mV
EDX	Elemental composition	Ag (major), C and O (minor)
FTIR	Major peaks (cm^-1^)	∼3,285, ∼1,636

**FIGURE 3:**
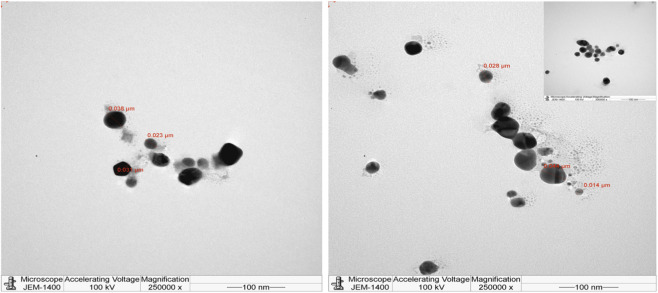
TEM images of G-AgNPs reveal predominantly spherical nanoparticles with smaller core sizes compared to DLS measurements. Data are presented as mean ± SD from three independent mesaurements. Scale bar = 100 nm at 250000 X magnifications.

In addition, the size, dissemination, and morphology of the biogenic NPs were quantitatively measured using TEM analysis (Figure 3). Well-dispersed and mostly spherical NPs (14–44 nm) were obtained with no some degree agglomeration. Similar-sized plant-synthesized NPs have been reported; TEM analysis on NPs synthesized with *Cuphea carthagenensis* leaf extract revealed spherical AgNPs (Rather et al., 2022). In the present study, some quasi-spherical AgNPs were also formed, which could be a common phenomenon in the bio-fabrication of NPs. Various shapes of biogenic NPs can also be formed, as was observed by Sreelekha et al. (2021) when preparing AgNPs with the leaf extract of *Mussaenda frondosa*. An earlier study on AgNPs prepared with *G. rigens* reported spherical and well-distributed NPs detected by SEM analysis and Ag atoms at 2.5 to 3.5 KeV recorded by the EDX spectrum (Shahzadi et al., 2021).

**FIGURE 4:**
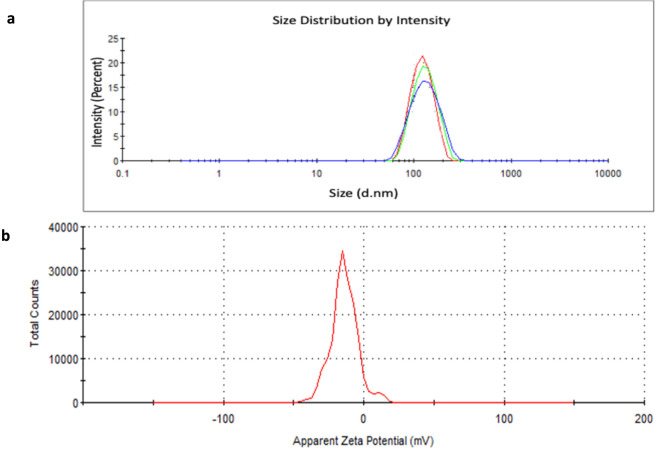
Dynamic light scattering (DLS) analysis of biogenic G-AgNPs indicating average hydrodynamic diameter of 135.4 nm with a polydispersity index (PDI) of 0.223, showing a moderately narrow size distribution. Different colors indicate the average size distribution of G-AgNPs from three different readings **(a)** and zeta potential **(b)**.

Further size assessment of the biogenic G-AgNPs in solution was performed using DLS, which showed an NP size of 135.4 nm with a polydispersity index (PDI) of 0.223 (Figure 4a; Table 1). This size is notably larger than that obtained by TEM analysis, which reflects the physical core dimensions of dried nanoparticles imaged. DLS measurements are conducted in an aqueous medium and determine the hydrodynamic diameter, which includes the metallic core together with the surrounding solvation layer and phytochemical capping molecules derived from the plant extract (Bhattacharjee, 2016; Fan et al., 2014). Furthermore, because scattering intensity strongly depends on particle size, the DLS data were recorded as intensity-weighted distributions, which are intrinsically skewed toward larger particles or ephemeral aggregation. As a result, the measured diameter may be significantly affected by even slight aggregation or clustering in solution. Mohammed et al. (2022) and Rizwana et al. (2022) also reported for green-synthesized nanoparticles, similar differences between TEM and DLS particle sizes. Furthermore, because DLS measurements are very sensitive to surface charge and capping effects, the presence of surface-bound phytochemicals and the corresponding electrical double layer have a considerable impact (Bamal et al., 2021). Furthermore, the characteristics of bio-fabricated NPs are also affected by their PDI. The PDI value obtained in the current study was below 0.3, which indicates the formation of a monodispersed form of the G-AgNPs (Manosalva et al., 2019). Zeta potential analysis indicated −13 mV (Figure 4b), signifying moderate colloidal stability, which may permit partial aggregation. The observed negative charge is apparently due to phyto-molecules that reduced and covered the biogenic NPs, thereby preventing adherence (Mohammed and Al-Megrin, 2021). Qualitative agreement between TEM and DLS measurements was observed.

**FIGURE 5:**
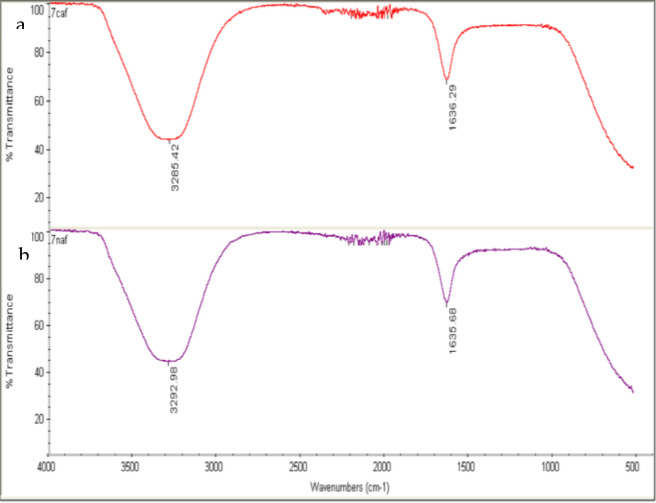
FTIR spectra of **(a)** *G. rigens* extract and **(b)** biogenic silver nanoparticles (G-AgNPs). The broad absorption bands observed at ∼3,285–3,293 cm^-1^ correspond to O–H stretching and peaks around ∼1,635–1,636 cm^-1^ correspond to C=O/amide I.

The *G. rigens* leaf aqueous extract and the G-AgNPs were subjected to FTIR analysis to identify the phytochemical functional moieties in the plant extracts involved in the bio-reduction process. High absorbance peaks at 3,285.42 and 1,636.29 cm^-1^ were noted for the G-AgNPs, and 3,292.98 and 1,635.68 cm^-1^ for the leaf extract (Figure 5). Several inorganic and organic compounds were detected by FTIR analysis. Bands detected at 1,620–1,680 cm^-1^ might be linked to C=C (Alkenyl stretch), and the bands at 3,200–3,400 cm^-1^ could demonstrate H-bonded OH stretch, Hydroxy group (Nandiyanto et al., 2019). The magnitude of the bands showed slight differences between the two tested agents, which could indicate the utilization of phytochemicals in the AgNP fabrication. The presence of the same functional group, such as OH in G-AgNPs, suggests that organic molecules from the plant extract reduced and formed a capping layer covering the NPs, acting as a nano-sized layer that protected them from sedimentation and aggregation (Alahmad et al., 2022). The interaction of plant extract enzymes with Ag ions forms an enzyme-substrate complex, enabling charge transfer between Ag^+^ and the phytochemical, leading to the production of protein-capped AgNPs (Jain and Mehata, 2017). The inherent cytotoxicity of silver nanoparticles is mitigated by using *G. rigens* extract as a biogenic reducing and capping agent, which not only promotes AgNP formation but also improves biocompatibility (Eker et al., 2025; Simon et al., 2022).

### 3.2 The antibacterial efficacy of G-AgNPs

The antibacterial efficacy of G. *rigens* leaf extracts and G-AgNPs against *S. mutans*, *S. aureus*, *E. coli*, and *K. pneumoniae* was tested. In addition, the combination of ampicillin, a broad-spectrum antibiotic, with G-AgNPs was studied to identify any additive or synergistic therapeutic effects against the tested microbes. This combination is of particular interest because it may enhance the antibacterial activity of ampicillin, which could be recommended especially against ampicillin-resistant strains.

**FIGURE 6:**
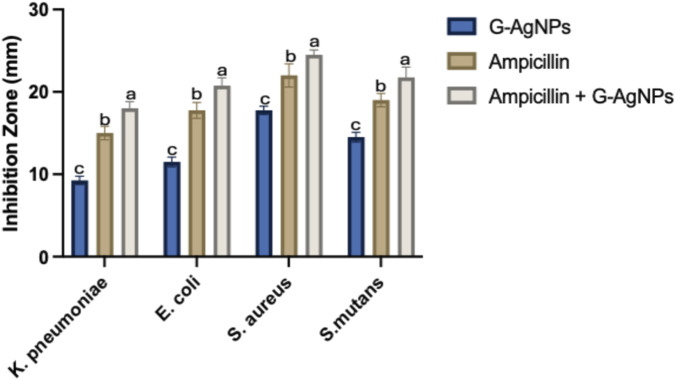
G-AgNPs, ampicillin, and ampicillin-associated G-AgNPs (ampicillin + G-AgNPs) were tested against four bacterial strains. Data are means and ± SD of the inhibition zones (mm) for three replicates. Significant variations (*P* < 0.001) among the tested agents were noted and indicated with different letters.

The leaf extract of *G. rigens* alone had no antibacterial activity; however, it is essential to note that a low concentration was tested. The inhibition zone for G-AgNPs ranged between 9.25 ± 0.5 and 17.75 ± 0.5; for ampicillin, it was between 15.00 ± 0.8 and 22.00 ± 1.4, and the highest zone for the amp-G-AgNP conjugate was between 18 ± 0.8 and 24.5 ± 0.5 mm (Figure 6). The multiple comparison tests indicated significant variations in the responses of each bacterium to the different agents (G-AgNPs *P* < 0.0001, ampicillin *P* < 0.0001 and ampicillin + G-AgNPs *P* < 0.001). Interestingly, a similar response was noted for all tested agents; however, *K. pneumoniae* was tolerant, and *S. aureus* was more sensitive than others. The enhanced antibacterial activity observed for the ampicillin-AgNP formulation could be attributed to co-dispersion and surface association effects, rather than confirmed chemical conjugation.

The results revealed that MICs for all tested strains ranged from 0.062 to 0.125 mg/mL, and MBCs ranged from 0.125 to 0.250 mg/mL (Table 2). The MBC values for all tested strains were higher than the MIC values. Similar observations were noted in a previous study when twelve antimicrobials were tested against eight Strains of *Listeria monocytogenes* (Rodríguez-Melcón et al., 2021). In the current study, the tolerance level (MBC/MIC) showed that the tested strains were non-tolerant (CLSI, 2016). A similar finding was observed when bacterial strains were treated with AgNPs prepared by fungal extracts as biogenic agents (Sonbol et al., 2022). Extensive reports highlight the ability of nano-antibiotics to effectively disrupt and mitigate microbial biofilms due to the physical interaction between them, which is anticipated to enhance their efficacy, especially in clinically relevant settings such as lungs and wounds (Siemer et al., 2019). The application of AgNPs could enhance ROS production, thereby damaging microbial cells (Kemala et al., 2022; Wypij et al., 2021). A high contact surface area of the NPs also supports greater stability and biological activities (Ramos et al., 2022). The small size of Ag-NPs may be a key factor in their ability to prevent biofilm formation (Murray et al., 2022). Their tiny dimensions enable them to effectively reach and interact with bacterial cells, altering the bacterial cell wall and membrane. This interaction enables them to penetrate the cell, inhibit enzymes, and disrupt DNA. Additionally, the accumulation of Ag-NPs within cells can affect several vital functions by altering cell structure and transport activities (Aabed and Mohammed, 2021a; Dakal et al., 2016; Prabhu and Poulose, 2012; Yin et al., 2020).

**TABLE 2:** MICs and MBCs for the G-AgNPs.

Bacteria strains	MICs (mg/mL)	MBCs (mg/mL)
*K. pneumoniae*	0.125	0.250
*E. coli*	0.062	0.125
*S. aureus*	0.062	0.125
*S.mutans*	0.062	0.125

MIC and MBC values represent the lowest concentration inhibiting visible growth (MIC) or resulting in no recoverable growth (MBC), consistently observed across three independent experiments.

Recent study reported that standared antibiotics often show variable and limited efficacy against resistant clinical isolates (Zhuang et al., 2023), however, combination strategy of therapy could have a potential impact in improving the antibacterial effect of one antibiotic (Yang et al., 2025). Currently, G-AgNPs association increased the activity of ampicillin against all tested microbes; a similar partial additive effect was also reported earlier with *Malvaviscus arboreus* green-synthesized AgNPs conjugated with ampicillin tested against the same bacteria (Mohammed et al., 2023). However, in the case of *Jatropha integerrima* AgNPs, conjugation with ampicillin had a partial antagonistic effect on *S. aureus* and *K. pnuemoniae* and a complete antagonistic effect on *S. mutans* with no impact on the efficacy in *E.coli* (Mohammed et al., 2021). Thus, the choice of plant extract is an important determinant for biological activity; each plant source produces distinctive results. Typically, when biogenic AgNPs are combined with antibiotics such as ampicillin, their biological activity is augmented relative to their individual effects, as the antibiotic coats the surfaces of AgNPs (Khurana et al., 2021). Mba and Nweze, (2021) indicated that the antibacterial effects of nanoparticles could be significantly improved when conjugated with antibiotics, as they act as carriers for such materials that facilitate their entry into cells, leading to cell damage. Ampicillin targets the bacterial cell wall and, therefore, could facilitate the entry of NPs into the cell, allowing targeting of cellular components (Egorov et al., 2018). Several studies have found that antibiotics significantly enhance the antimicrobial activity of AgNPs when conjugated. However, we observed only a weak, partial additive antimicrobial effect. A plausible limitation influencing the experimental results is the efficacy threshold, which could have led to the partial additive response seen with the ampicillin-associated AgNPs treatment. Ensuring that a ceiling effect does not contribute to the observed results is essential, and one way to do this is to repeat the experiments using different concentrations of G-AgNP and amoxicillin (Masri et al., 2018; Naqvi et al., 2013). Furthermore, spectroscopic and colloidal stability studies are essential to confirm physicochemical behavior of ampicillin-associated G-AgNPs.

### 3.3 The cytotoxic potential of G-AgNPs

**FIGURE 7:**
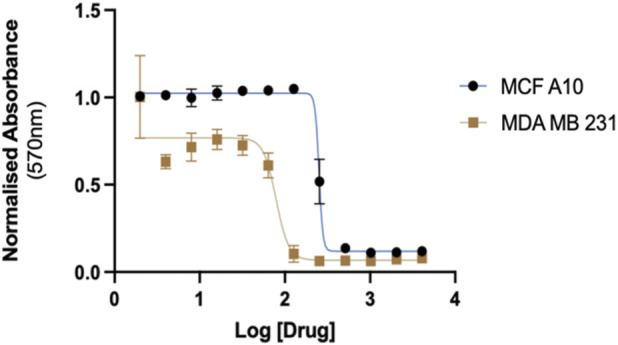
G-AgNPs cytotoxic effect on breast cancer cell line (MDA-MB-231) and a non-cancerous breast epithelial cell line (MCF-10A). Figure shows the log dose-response to derive the IC_50_.

The G-AgNP cytotoxicity was tested against the two human breast cell lines, MDA-MB-231 (cancer) and MCF-10A (normal), using the MTT assay. Treating the cells with increasing concentrations of G-AgNPs (0–4,080 μg/mL) resulted in a dose-dependent decrease in cell viability. The IC_50_ values were calculated using GraphPad Prism: 78.66 μg/mL for MDA-MB-231 and 251.8 μg/mL for MCF-10A (Figures 7a,b). The positive control Mitoxantrone indicated an IC_50_ of (0.32 μg/mL). The results showed that G-AgNPs were significantly (*P* < 0.0001) potent at killing the breast cancer cell line than the normal breast epithelial cell line, indicating an acceptable selectivity ratio for IC_50_ for normal cells and IC_50_ for cancer cells. As such, G-AgNPs might be valuable for further research and development as a nanomaterial with the potential to treat breast cancer. Reports showed that other plants, such as *J. integerrima* and *M. arboreus*, green-synthesized AgNPs, had almost equal cytotoxic potency in cancerous and non-cancerous cells (Mohammed et al., 2021; Mohammed et al., 2023). Hence, our initial results with G-AgNPs are promising and warrant further screening of cancerous and non-cancerous cells to establish any tangible therapeutic value. The anti-proliferative and cytotoxic potential of various bio-fabricated AgNPs against the same cell lines has been investigated previously (Mohammed and Al-Megrin, 2021; Mohammed et al., 2021). The MTT assay in the current study reflects cellular metabolic activity and does not distinguish between apoptosis and growth inhibition. As microscopy-based imaging and apoptosis assays were not performed, the observed effects are reported as general cytotoxicity. Based on prior literature, one potential mechanism by which nanoparticles can cause cellular damage upon interaction with cells is the induction of oxidative stress, which generates ROS (Roy et al., 2019; Xu et al., 2020). The involvement of ROS in inducing cell apoptosis has been validated in studies where breast cancer cell lines were exposed to nanoparticles (Gill et al., 2016; Mahalingaiah and Singh, 2014). Furthermore, NPs could induce cell apoptosis through DNA damage and mitochondrial dysfunction (Gurunathan et al., 2018), as well as the diffusion of cell organelles (Mohammed et al., 2021). According to a study by Kalishwaralal et al. (2009), AgNPs have been shown to affect the activity of vascular endothelial growth factor (VEGF), which plays a significant role in tumor angiogenesis. The small size of nanoparticles significantly contributes to their high affinity for cells, which may be a primary factor in their interaction with cellular structures, causing ultrastructural modifications and cellular damage. Kepsutlu et al. (2020) reported the role of phyto-molecules that cover the NPs in enhancing their compatibility with the biological system. Since AgNPs induced mammalian cell death, they could be promising therapeutic agents. Their mode of action exhibits certain similarities to that of other chemotherapeutic agents. There are other reports of biosynthesized AgNPs being more selective to cancerous cells than non-cancer cells. One such study on Soybean agglutinin AgNPs showed that the AgNPs were cytotoxic against breast cancer cell lines and had no activity against non-cancerous breast epithelial cells (Casañas Pimentel et al., 2016). One possible mechanism of greater efficacy in cancerous cells may be enhanced accumulation, which can occur passively or be targeted (Kovács et al., 2022). In the case of green-synthesized NPs, our previous studies indicated targeted accumulation occurs via the phytochemicals making up the external layer of the NPs, leading to ultrastructural changes for the cancer cells treated with biogenic NPs compared to untreated control (Mohammed and Al-Megrin, 2021; Mohammed et al., 2021).

Furthermore, elevated metabolic activity of cancer cells and greater fluidity of the cell membrane of cancer cells may also contribute to the accumulation and interaction of AgNPs. Aggressive cancers have mesenchymal-type properties with distinct microenvironments and cellular interactions. AgNPs are known to enter mesenchymal cells readily through clathrin-dependent endocytosis and micropinocytosis (Zhang et al., 2016). As such, AgNPs may selectively accumulate in cells and tissues, dependent on key cellular features such as cellular metabolic activity, motility, functionality, communication, and signaling. Hence, further research is required to fully understand the nature of AgNP interactions and the degree of accumulation in distinct cell and tissue types.

## 4 Conclusion

Given the growing threat of drug-resistant pathogens and the limitations of conventional chemotherapy and antibiotics, the search for novel therapeutic agents has become imperative. In this study, G. rigens leaf extract was demonstrated to function as an effective green bioreductant for the synthesis of silver nanoparticles (G-AgNPs), yielding predominantly spherical and monodispersed with some degree of agglomerated particles. Under *in vitro* conditions, the synthesized nanoparticles exhibited antibacterial activity against selected Gram-positive and Gram-negative bacterial strains, including *S. aureus*, *S. mutans*, and *E. coli*. In addition, G-AgNPs showed cytotoxic effects against breast cancer cells, while displaying comparatively lower toxicity toward normal epithelial breast cells, suggesting a degree of cellular selectivity in this experimental model. While these findings indicate the potential biomedical relevance of G-AgNPs, the present study is limited to *in vitro* evaluations using a single cancer cell model and does not include *in vivo* validation or comprehensive mechanistic investigations. Consequently, the translational significance of these results remains preliminary. Future studies should therefore focus on expanding mechanistic analyses, validating efficacy and safety in appropriate *in vivo* models, and assessing pharmacokinetic and toxicological profiles to better define the applicability of green-synthesized AgNPs in biomedical contexts.

## Data Availability

The raw data supporting the conclusions of this article will be made available by the authors, without undue reservation.
